# A Simple Method of Relieving Retention of Urine in Disease of the Prostate Gland, or in Injury of the Urethra, When Hæmorrhage from Either the Prostate or Lining Membrane of the Urethra Takes Place

**Published:** 1848-11

**Authors:** M. C. Bernard

**Affiliations:** Medical Superintendent to the Dundrum Dispensary, County Dublin


### RECORD OF MEDICAL SCIENCE.

*A simple method of relieving retention of Urine in disease of the Prostate Gland, or in injury of the Urethra, when Hæmorrhage from either the prostate or lining Membrane of the Urethra takes place.* By M. C. Bernard, M. B., L. R. C. S. I., &c., Medical Superintendent to the Dundrum Dispensary, County Dublin.—It is well known to every practical surgeon, the distress which patients labouring under disease of the prostate gland constantly suffer from retention of urine. This retention, which is for the most part caused by mechanical obstruction, (the walls of the urethra being either close together by the swollen prostate, on the one hand, or the third lobe overlapping in a valvular manner the entrance of the urinary passage on the other,) may often be easily removed by a manœuvre first suggested by Mr. Hey, and now well known to every surgeon—viz., by withdrawing the stilet when the instrument has passed the membranous portion of the urethra, whereby the top of the catheter is tilted into the bladder. Leeches to the perineum, the hip-bath, and anodyne enemata, are also useful auxiliaries to allay the inflammation and irritation which generally attends on these cases.

There is not, perhaps, a gland in the body more susceptible to changes in volume from atmospheric influences, than the diseased prostate. If a patient, who is afflicted with this complaint, gets even an ordinary cold, he is sure to suffer from an aggravation of all his symptoms. The prostate gland, already in a state of hypertrophy, becomes inflamed and congested: sometimes a permanent varicose state of the gland may supervene. In this altered state of the parts, the walls of the urethra are so closely brought in contact, that the patient with all his efforts cannot pass one drop of urine; often, in his vain efforts to micturate, the overloaded vessels of the distended prostate will give way, and hæmorrhage to a considerable extent may take place either into the urethra or bladder.

Under circumstances of this description, there is often considerable difficulty in passing an instrument into the bladder, and when the surgeon succeeds in introducing the ordinary gum-elastic catheter, he may find to his disappointment, after withdrawing the stilet, that not one drop of urine will pass: in fact, the instrument becomes choked with coagulated blood. Exactly the same thing may occur after you have succeeded in passing an instrument into the bladder, either in rupture of the urethra from accident, or where a false passage has been made by a forcible attempt to introduce a catheter.

The means I successfully adopted under a similar difficulty may be best understood by the narration of the following case.

On the 21st of April last, I was called on to see Captain P., ætat. 64, labouring under retention of the urine, with diseased prostate: he was suffering great pain. Having allowed the water to accumulate for upwards of thirty-six hours, obtaining occasionally a little relief by the urine passing in drops, I introduced a large sized gum-elastic catheter into the bladder with the greatest ease, and drew off nearly two quarts of urine of a dark chocolate colour, which afforded instantaneous relief. As my patient lived at a distance, I was unable to see him more than once or twice a day; and as the calls to make water were frequent, I instructed his son—an intelligent young man—in the mode of using the instrument. After a few days a gradual amendment took place in all the symptoms. Early on the morning of the 12th of May, my immediate assistance was again requested. The son informed me that his father had got cold on the previous evening; that he could not succeed in passing the instrument into the bladder; and that after each attempt, the instrument was filled with blood. When I saw my patient, he was pale, shivering, and in intense agony, the whole body being bedewed with a cold perspiration. He implored to be relieved immediately from his distress. After a little manœuvring I passed the ordinary full-sized gum-elastic catheter into the bladder: not one drop of urine, however, would flow, and upon withdrawing the instrument, I found it blocked up with coagulated blood. As the symptoms were now of an urgent nature, I sent off a messenger for a syringe, with the intention of injecting tepid water into the bladder and dislodging the blood from the instrument. In the mean time I thought of a simple expedient: I took a gum-elastic catheter of large calibre, without its stilet, and introduced into it (in the same manner you would put your finger into a glove) another catheter armed with its stilet, and of sufficfent size to fill completely the larger one. I passed this double catheter with the greatest freedom into the bladder, and had the happiness to find upon withdrawing the smaller one, that the urine flowed in a full stream, at the same time giving my patient immediate relief from his protracted suffering.

I have brought forward this case principally with the view of showing how little dependence ought to be placed, especially in cases of emergency, in the ordinary gum-elastic catheters, which are sold at the surgical instrument makers. At a time when so much ingenuity is shown in the improvement of the lithotrite, surely the gum-elastic catheter—an instrument in such constant use—ought to receive some attention and care in its manufacture. Those in ordinary use are accompanied with stilets, which do not half fill up the catheter: in some, the stilet does not go even to the end of the instrument: in others, on the contrary, it is too long, and possesses a resilience which causes it to rebound from the catheter when it commences its circuit under the arch of the pubes, so that unless great caution is used in attempting to adjust it, the stilet may be thrust through the eye of the instrument and lacerate the urethra. To avoid accidents of this nature, the stilet should be made to fill the catheter completely. The late Surgeon Colles, in his lectures published in the Medical Press, vol. xii. No. 288, in speaking of disease of the prostate gland, says—"If you must use a stilet, let it be an inflexible one, and one that fills the catheter completely: one made of brass is best." Surgeon Wilmot, in his lectures on the same subject, and published in the same invaluable journal, advocates the same principle. He says—"I was not long in practice when I found the inutility of the slender and flexible stilets which are sold along with the instruments."

The double catheter which I employed in the above mentioned case, independent of its preventing coagulated blood from blocking up its cavity, appeared to have another great advantage over the ordinary catheter. It seems to glide into the bladder with greater ease, and to cause less pain and irritation. I can account for this circumstance in one way only—that this double instrument combines within itself the properties of both the bougie and catheter, and possesses the softness of the one as well as the inflexibility of the other.

In the construction of the large sized gum-elastic catheter, I am of opinion that both of these indications may be fulfilled, simply by making the walls of the catheter (which are composed of web silk) of much thicker consistence, at the same time that its brass stilet should be carefully made to fill completely its cavity.

A catheter made in this way will glide into the bladder with great ease and freedom, and there is no possibility of its cavity becoming obstructed with coagulated blood.—*Dub. Med. Press.*

